# Prevalence and Predictors of Left Ventricular Diastolic Dysfunction in Malaysian Patients With Type 2 Diabetes Mellitus Without Prior Known Cardiovascular Disease

**DOI:** 10.3389/fcvm.2021.676862

**Published:** 2021-09-27

**Authors:** Kok Han Chee, Kok Leng Tan, Ibrahim Luqman, Shahrizal Shudim Saiful, Yee Yean Chew, Karuthan Chinna, Alexander Tong Boon Tan

**Affiliations:** ^1^Medical Department, University Malaya Medical Centre, Kuala Lumpur, Malaysia; ^2^Regenerative Medicine Cluster, Institut Perubatan dan Pergigian Termaju, Universiti Sains Malaysia, Kepala Batas, Malaysia

**Keywords:** diastolic dysfunction, diabetes mellitus, left ventricular dysfunction, prevalence, Asian

## Abstract

**Objective:** Existing data showed that left ventricular diastolic dysfunction is common in individuals with type 2 diabetes mellitus (T2DM). However, most of the studies included diabetic patients who have prior cardiovascular disease, which might be the compounding factor for ventricular dysfunction. This study aimed to determine the prevalence and predictors of left ventricular diastolic dysfunction in an Asian population with T2DM without prior cardiovascular disease using the latest recommended echocardiographic assessment for left ventricular diastolic dysfunction.

**Design and Participants:** This is a cross-sectional study in which eligible patients with T2DM without history of coronary artery disease, heart failure, or valvular heart disease were recruited. Demographic data, diabetic control, comorbidities, microvascular/macrovascular complications, and medications prescribed were recorded. Venous blood was sent to test for B-type natriuretic peptide, and transthoracic echocardiography was performed to assess left ventricular dysfunction.

**Setting:** This study was performed in a tertiary healthcare center located in Kuala Lumpur, Malaysia.

**Results:** Of the 301 patients, 83.1% have had T2DM for >10 years, with 45.8% being poorly controlled. Comorbidities include hypertension (77.1%), hyperlipidemia (91.0%), and pre-obesity/obesity (72.9%). Majority had absence of microvascular (albuminuria, retinopathy, and neuropathy) and macrovascular (peripheral vascular disease and stroke) complications. None had raised B-type natriuretic peptide levels, and 93.7% had no symptoms of heart failure. On echocardiographic assessment, 70.1% had left ventricular diastolic dysfunction, and 90.5% had Grade 1/mild severity. Age, ethnicity, insulin therapy, presence of hypertension, and hyperlipidemia were significantly associated with left ventricular diastolic dysfunction. Older T2DM patients of Chinese ethnicity and on insulin are about two times more likely to develop left ventricular diastolic dysfunction.

**Conclusion:** There was a high prevalence of asymptomatic left ventricular diastolic dysfunction among patients with T2DM without prior known cardiovascular disease. Older age, insulin therapy, and Chinese ethnicity were risk factors for left ventricular diastolic dysfunction in T2DM.

## Strengths and Limitations

- This was the first study reporting the prevalence and predictors of left ventricular diastolic dysfunction (LVDD) in a multiethnic Southeast Asian type 2 diabetes mellitus cohort without prior known cardiovascular disease.
- Two-dimensional echocardiographic assessment for LVDD using the latest ASE/EACVI 2016 recommendations.
- This study did not definitively exclude coronary artery disease using coronary angiogram.

## Introduction

Heart failure (HF) is an important cause of morbidity and mortality in type 2 diabetes mellitus (T2DM) (1). The Framingham Heart Study revealed that HF is more prevalent in individuals with diabetes when compared to those without, with a five-fold increase in women and a two-fold increase in men (2). The presence of T2DM is also associated with worse clinical status and higher all-cause and cardiovascular (CV) mortality in both reduced and preserved ejection fraction HF (3). Unlike left ventricular systolic dysfunction (LVSD), left ventricular diastolic dysfunction (LVDD) is often underdiagnosed in diabetes. LVDD is itself associated with poor prognosis and can progress to LVSD (4, 5) and is thought to predate the onset of LVSD in patients with diabetes.

The American Society of Echocardiography (ASE)/European Association of Cardiovascular Imaging (EACVI) 2016 (6) recommendations introduced an uncomplicated method to diagnose and grade the severity of LVDD. Echocardiographic parameters such as left atrium volume index, tricuspid regurgitation velocity, E/e′, and septal or lateral e′ velocity were used for the diagnosis of LVDD in the presence of normal left ventricular ejection fraction (LVEF), whereas the diagnosis of HF with preserved ejection fraction (HFpEF) incorporates clinical signs and symptoms of HF, presence of LVDD on echocardiography (ECHO), and rise in biomarkers [B-type natriuretic peptide (BNP)].

The Strong Heart Study demonstrated T2DM to be independently associated with asymptomatic LVDD (1). However, it is important to note that many previous studies were conducted in a CV diabetic cohort and before the widespread use of renin–angiotensin system (RAS) blockers and statins or the advent of glucose-lowering drugs such as sodium-glucose co-transporter-2 inhibitors (SGLT2-i) or glucagon-like peptide-1 receptor analog (GLP1-RA) that modify risk of ischemic heart disease (IHD) and HF. Therefore, the prevalence and predictors of LVDD in populations using the latest guideline-directed medical therapy may differ.

Most data on HF in individuals with T2DM have been derived from Caucasian populations with limited studies in Asian patients. There may be differences in the clinical features, prevalence, and predictors of HF in Asian ethnicities. It is well-known that migrant South Asians are at higher risk of coronary artery disease compared with European Caucasians living in the same environment (7). Therefore, it is possible that Asian ethnicities may have differences in risk of HF as well. The few published studies evaluating Asian ethnic groups involve migrant populations living in the West who are potentially exposed to diets, degrees of physical activity, and socioeconomic factors that differ from those in their countries of origin. Also, these analyses sometimes failed to view Asians as a heterogeneous group and analyzed different Asian ethnic subgroups as a single entity. Emerging evidence shows that Singapore Asians (63% Chinese, 26% Malay, and 11% Indian) with HF, when compared to New Zealand Caucasians, are at lower risk of atrial fibrillation, especially if they have T2DM (8). Comparing data from population-based HF cohorts, Bank et al. (9) found that Southeast Asians with HF have a three-fold higher prevalence of diabetes compared with European Caucasians despite being younger and less obese. Asian patients with T2DM with HF also had poorer outcomes such as increased all-cause mortality compared to their Caucasian counterparts (9). While these findings are of great interest, that study analyzed three Asian ethnicities (Chinese, Malay, and Indian) as a single “Asian entity.”

Early detection of LVDD can lead to the institution of preventive measures to halt disease progression. There is a need to delineate the prevalence and predictors of diastolic dysfunction to enable systematically targeted intervention aimed at reducing morbidity and mortality in patients with T2DM. We designed this study to determine the prevalence of LVDD in the current landscape of T2DM management, where RAS blocker and statin use is more widespread. LVDD was diagnosed using echocardiographic parameters based on the latest ASE/EACVI 2016 recommendations in a multiethnic population of Malaysians with T2DM without known HF, coronary artery disease, or valvular heart disease. We also evaluated the strength of association of clinical predictors, in particular glycemic control, and ethnicity with the presence of LVDD.

## Materials and Methods

### Study Design and Participants

This was a cross-sectional study conducted at the University Malaya Medical Center (UMMC), a tertiary healthcare center located in the city of Kuala Lumpur, Malaysia. The prevalence of LVDD was estimated to be 50% in a diabetic population (10). A sample size of 271 was required with 5% absolute precision and 90% confidence for single-proportion estimation. The study was conducted between January and December 2018. The study protocol was approved by the institutional ethical review board (UMREC ID NO 20171126-5850) and fully conformed to the principles of the Helsinki Declaration.

All patients with T2DM who visited the diabetic outpatient clinic during the study period were assessed for eligibility in participating in this study. Written informed consent was obtained from all participants. Patients with known history of coronary artery disease, HF, valvular heart disease, and end-stage renal failure requiring dialysis were excluded. Ineligibility also extended to those with malignancy, acute infection, or inflammatory disease.

Demographic data included age, gender, ethnicity, weight, and height, and the presence of comorbidities such as hypertension and hyperlipidemia was recorded. Body mass index (BMI) categorization was done based on the World Health Organization Expert Consultation on Asian BMI report. The evaluation of diabetes and its complications included the duration of T2DM, HbA_1c_ status, renal function [presence of albuminuria and estimated glomerular filtration rate (eGFR)], and presence of other macrovascular and microvascular complications (stroke, peripheral vascular disease, retinopathy, and neuropathy). Current prescribed medications for all patients were recorded.

### Evaluation of Left Ventricular Dysfunction

Evaluation of HF in all patients was done by taking a detailed history and performing clinical examination, ECHO, and venous blood sampling for BNP level.

The history of HF symptoms as defined by the New York Heart Association (NYHA) Functional Classification (11) was elicited from all patients. Symptoms elucidated include level of breathlessness, fatigue, and palpitation related to activity; presence of orthopnea; and occurrence of paroxysmal nocturnal dyspnea (PND). Clinical examination of the CV system including presence of pedal edema, raised jugular venous pressure (JVP), and the respiratory system for signs of HF.

Transthoracic ECHO was performed by a senior sonographer who was blinded to the study outcome. Echocardiographic parameters such as left atrium volume index, tricuspid regurgitation velocity, E/e′, and septal or lateral e′ velocity were recorded. LVDD was classified and graded according to the latest recommendations of the ASE/EACVI 2016. LVSD was defined as an ejection fraction of <50%. Those with structural abnormalities (previously undiagnosed) were excluded from the study.

The evaluation of BNP levels was done by analyzing plasma BNP using Siemens ADVIA® Centaur BNP assay, which is an automated two-site sandwich chemiluminescent immunoassay.

### Statistical Method

Data were analyzed using SPSS version 22. Descriptive statistics were used for analysis of demographic characteristics. Continuous variables were expressed as mean ± SD. The differences between normally distributed numeric variables were evaluated by *t*-test or one-way analysis of variance, while non-normally distributed variables were analyzed by Mann–Whitney *U*-test or Kruskal–Wallis variance analysis, as appropriate. Multivariable logistic regression analysis was done to identify the association of independent variables.

### Patient and Public Involvement

There was no patient and public involvement in the design, conduct, reporting, or dissemination plans of our research.

## Results

### Patient Population

Three hundred and fifty patients with T2DM were screened during the study duration, and 315 were included in the study. However, 14 patients were excluded from the final analysis, as they did not proceed with blood investigations. The remaining 301 patients had blood investigations and ECHO assessment for LVD. The demographic characteristics are listed in Table 1.

**Table 1:** Demographic characteristics.

**Variable**	***n* (%)**
**Age**	
Mean (min, max)	61 (26, 86)
**Gender**	
Male	106 (35.22)
Female	195 (64.78)
**Ethnicity**	
Malay	85 (28.24)
Chinese	87 (28.90)
Indian	123 (40.86)
Others	6 (2.00)
**Obesity**	
Underweight	7 (2.34)
Normal	34 (11.37)
Overweight	40 (13.38)
Pre-obese	122 (40.80)
Obese	96 (32.11)
**Comorbidities**	
Hypertension	
Present	232 (77.08)
Absent	69 (22.92)
**Hyperlipidemia**	
Present	274 (91.03)
Absent	26 (8.64)
No data	1 (0.33)

*Total number of patients evaluated were n = 301 except for obesity, n = 299. Obesity was categorized based on the World Health Organization Expert Consultation on Asian Body Mass Index (12) (kg/m^2^): underweight, <18.5; normal, 18.5–22.9; overweight, 23.0–24.9; pre-obese, 25.0–29.9; and obese, ≥30.0*.

### Evaluation of Diabetic Status

The diabetic status of the patients is listed in Table 2. Most patients (83.1%) had T2DM for >10 years. The mean HbA_1c_ was 8.3% (±1.7%) with almost half (45.8%) being poorly controlled (HbA_1c_ > 8.0%). Majority had absence of microvascular (albuminuria, retinopathy, and neuropathy) and macrovascular (peripheral vascular disease, and stroke) complications.

**Table 2:** Evaluation of diabetic status including diabetic complications.

**Variable**	***n* (%)**
**Duration of diabetes (years)**	
<5	21 (6.98)
5–10	30 (9.97)
>10	250 (83.05)
**HbA**_**1c**_ **(%)**	
Mean (min, max)	8.3 (5.4, 15.5)
<7	61 (20.27)
7–8	102 (33.89)
>8	138 (45.84)
**Renal function**	
Albuminuria	
Present	86 (28.57)
Absent	215 (71.43)
**eGFR (ml/min/1.73 m** ^**2**^ **)**	
Mean (min, max)	78 (24, 110)
Normal (≥90)	156 (51.83)
Mild (60–89)	100 (33.22)
Moderate (30–59)	44 (14.62)
Severe (<30)	1 (0.33)
**Retinopathy**	
Present	67 (22.26)
Absent	234 (77.74)
**Neuropathy**	
Present	58 (19.27)
Absent	243 (80.73)
**Peripheral vascular disease**	
Present	6 (2.01)
Absent	293 (97.99)
**Stroke**	
Present	16 (5.32)
Absent	285 (94.68)

*Total number of patients evaluated were n = 301 except for peripheral vascular disease, n = 299; eGFR, estimated glomerular filtration rate*.

### Assessment of LVD

Of 301 patients, 19 (6.3%) presented with symptoms of HF (Table 3). Most patients were classified into New York Heart Association (NYHA) Class 1. Only 3.7% of patients had dyspnea on exertion while 0.3% complained of orthopnea. None had dyspnea at rest or PND. On clinical examination, only nine (3.0%) had pedal edema, and none had raised JVP.

**Table 3:** Assessment for LVD.

**Variable**	***n* (%)**
**Symptoms of HF**	
Present	19 (6.33)
Absent	281 (93.67)
**LVDD**	
Present	211 (70.10)
Absent	90 (29.90)
**Severity of diastolic dysfunction**	
Grade 1	191 (90.52)
Grade 2	18 (8.53)
Grade 3	2 (0.95)
**LVEF (%)**	
Mean (min, max)	68 (50, 78)
**BNP (pg/ml)**	
Mean (min, max)	25.81 (2, 206)

*n = 301 except for severity of diastolic dysfunction, n = 211; LVEF, left ventricular ejection fraction; BNP, B-type natriuretic peptide*.

On ECHO assessment, 211 patients (70.1%) had LVDD according to the ASE/EACVI 2016 recommendations. LVDD severity was mostly Grade 1 (90.5%). None of the patients exhibited LVSD (results not shown). The mean LVEF using the modified Simpson method was 68%.

Mean BNP was 25.81 pg/ml (±27.87) (range 2–206 pg/ml), and none of the patients had abnormal BNP level (results not shown).

### Medicines Prescribed

Metformin was the most prescribed glucose-lowering drug followed by insulin (Table 4). A similar proportion of patients was prescribed with SGLT2-i and dipeptidyl peptidase 4 inhibitors (DPP4-i), while the alpha-glucosidase inhibitor was the least used. Angiotensin-converting enzyme inhibitors (ACE-i) and angiotensin II receptor blockers (ARB) for hypertension were prescribed to 75.4% of the patients, while hyperlipidemia was treated mainly with statins (90.4%).

**Table 4:** Medications prescribed.

**Drugs**	**Yes, *n* (%)**	**No, *n* (%)**
**Glucose-Lowering agent**		
Metformin	276 (91.69)	25 (8.31)
Sulfonylurea	93 (30.90)	208 (69.10)
Insulin	181 (60.13)	120 (39.87)
SGLT2-i	126 (41.86)	175 (58.14)
DPP4-i	121 (40.20)	180 (59.80)
GLP1-RA	25 (8.31)	276 (91.69)
Alpha-glucosidase inhibitor	6 (1.99)	295 (98.01)
**Anti-hypertensives**		
ACE-i/ARB	227 (75.42)	74 (24.58)
Calcium channel blockers	137 (45.51)	164 (54.49)
Diuretics	53 (17.61)	248 (82.39)
β-Blockers	42 (13.95)	259 (86.05)
α-Blockers	15 (4.98)	286 (95.02)
Others	2 (0.66)	
**Lipid-Lowering agent**		
Statins	272 (90.37)	29 (9.63)
Fibrates	38 (12.62)	263 (87.38)
Ezetimibe	3 (1.00)	298 (99.00)
**Anti-platelets**	117 (38.87)	184 (61.13)
Aspirin	99 (32.89)	202 (67.11)
Clopidogrel	9 (2.99)	292 (97.01)
Ticlopidine	9 (2.99)	292 (97.01)
**Anti-coagulants**		
Novel oral anti-coagulants	1 (0.33)	300 (99.67)
Warfarin	0	301 (100)

*n = 301; SGLT2-i, sodium-glucose co-transporter 2 inhibitors; DPP4-i, dipeptidyl peptidase 4 inhibitors; GLP1-RA, glucagon-like peptide-1 receptor analog; ACE-i/ARB, angiotensin-converting enzyme inhibitors/angiotensin II receptor blockers*.

### Presence of LVDD Based on Clinical and Laboratory Characteristics

The mean age of patients with LVDD was 63 years (±9), older than patients without LVDD (Table 5). Indian (37.9%) and Chinese (34.1%) patients were slightly more affected. The proportion of patients with hypertension and LVDD was significantly higher (*p* = 0.006). Similarly, the number of patients with hyperlipidemia and LVDD was significantly higher than those without LVDD (93.84 vs. 86.67%; *p* = 0.039). Significantly more patients on insulin had LVDD (78.70 vs. 16.67%; *p* = 0.044).

**Table 5:** Presence of LVDD based on clinical and laboratory characteristics.

**Parameters**	**With LVDD**, ***n* = 211 (%)**	**Without LVDD**, ***n* = 90 (%)**	***p*-value**
Age, years ± SD	63 ± 9	57 ± 11	<0.001
Ethnicity			0.018
Malay	56 (26.54)	29 (32.22)	
Chinese	72 (34.12)	15 (16.66)	
Indian	80 (37.91)	43 (47.77)	
Others	3 (1.42)	3 (3.33)	
Gender			0.731
Male	73 (34.59)	33 (36.66)	
Female	138 (65.40)	57 (63.33)	
BMI, kg/m^2^ ± SD	27.37 ± 4.59	27.69 ± 5.35	0.596
Normal	68 (32.22)	21 (23.33)	0.302
Overweight	91 (43.12)	44 (48.88)	
Pre-obese and obese	52 (24.64)	25 (27.77)	
Smoking	17 (8.06)	6 (6.66)	0.914
Hypertension	172 (81.52)	60 (66.66)	0.006
Duration of hypertension			0.604
<5 years	5 (2.37)	1 (1.11)	
5–10 years	23 (10.90)	5 (5.56)	
>10 years	135 (63.98)	47 (52.22)	
Duration of T2DM			0.315
<5 years	12 (5.69)	9 (10.00)	
5–10 years	23 (10.90)	7 (7.78)	
>10 years	176 (83.41)	74 (82.22)	
Hyperlipidemia	198 (93.84)	78 (86.67)	0.039
Stroke	10 (4.74)	6 (6.67)	0.500
PVD	4 (1.90)	2 (2.22)	
Retinopathy	49 (23.22)	18 (20.00)	0.538
Neuropathy	41 (19.43)	17 (18.89)	0.913
Albuminuria	85 (40.28)	35 (38.89)	0.821
Microalbuminuria	53 (25.12)	19 (21.11)	
Macroalbuminuria	32 (15.17)	16 (17.78)	
Insulin therapy	166 (78.70)	15 (16.67)	0.044

*LVDD, left ventricular diastolic dysfunction; SD, standard deviation; BMI, body mass index; T2DM, type 2 diabetes mellitus; PVD; peripheral vascular disease*.

### Risk Association for LVDD

Age, ethnicity, insulin therapy, and presence of hypertension and hyperlipidemia were significantly associated with LVDD in our study. On univariate analysis, every unit of increase in age increased the risk of LVDD by 1.05 [95% confidence interval (CI) 1.03, 1.08; *p* < 0.001]. It also showed that hypertension increased the risk of LVDD by 2.21 (95% CI 1.26, 3.86; *p* = 0.006), but the duration and number of hypertensive medications did not. Besides, use of insulin increased the risk of LVDD by 2.41 (95% CI 1.27, 4.56; *p* = 0.007). On multivariate analysis, however, the presence of hypertension and hyperlipidemia lost its significance. Older T2DM patients of Chinese ethnicity and on insulin are about two times more likely to develop LVDD.

## Discussion

We found that none of our multiethnic high-risk Asian cohort with long-standing T2DM had evidence of LVSD on echocardiography. However, more than two-thirds of the patients had evidence of LVDD according to the ASE/EACVI 2016 recommendations (6), mostly at Grade 1/mild severity. Such low prevalence of LVSD, asymptomatic of HF cohort, is unexpected given that 86.3% of our patients were overweight/pre-obese/obese, 83.1% had been diagnosed with T2DM for more than a decade (60.1% insulin requiring), 77.1% had hypertension, and 91.0% dyslipidemia.

The remarkable absence of LVSD or higher grades of LVDD may be attributed to many of our patients being treated with statins (90.3%) and RAS blockers (75.4%), with as many as 41.9% receiving SGLT2-i. All these agents are known to modulate CV disease and HF. Our exclusion of patients with known heart disease, including coronary artery disease, which is the most important cause of HF in T2DM may account for such low prevalence. The widespread use of statins and RAS blockers may also explain the relatively low prevalence of retinopathy (22.3%) and albuminuria (28.6%) in our sample population.

We managed our patients at a government-funded tertiary healthcare center providing affordable care; it is uncertain whether our results could be extrapolated to primary and secondary healthcare settings. Nevertheless, compliance with international guidelines on standards of care and prescription of RAS blockers, statins, and SGLT2-i (13) can prevent or delay the progression of cardiac disease.

The prevalence of HF in patients with T2DM is reported to be 12–57% (14) while reports on the prevalence of LVDD in these patients vary from 23 to 75% (15). This variability accounted for the differences in diagnostic methods and patient cohorts (demographics, inclusion/exclusion criteria, and medications prescribed). A recent meta-analysis published in 2018 found the prevalence of LVDD to be 35.0% in the general T2DM population (16). There are only a handful of small studies (patient population <150) reporting a prevalence of LVD ranging 54.3–65.0% in Asian populations (17–19). Our observation that 70.1% of Malaysian patients with established T2DM on treatment have asymptomatic, mainly Grade 1/mild LVDD is not dissimilar to these reports. One study (18) reported that 92.2% of its LVDD patients had mild dysfunction. On the other hand, a study by Chaudhary et al. (20) on normotensive newly diagnosed T2DM patients before treatment initiation found an alarming 41.0% prevalence of LVDD, majority (87.8%) with Grade 1 dysfunction. Like these Asian studies, we found no patients with undiagnosed HF.

Some Western studies also found LVDD to be more prevalent than LVSD in patients without known coronary artery disease. In a cohort of 386 Italian patients (SHORTWAVE study) (1), 42% had confirmed LVDD (the majority with Grade 1 dysfunction) while 3.6% had EF < 50%. The mean age in this population was >60 years, with T2DM of short duration (mean of 4–5 years). Seventy-two percent were on RAS blockers and 45% on statins. The low prevalence of LVSD and LVDD in this study can be explained by the exclusion of inducible ischemia by stress echocardiography, short mean duration of T2DM, and widespread use of RAS blockers. However, in a cohort of Danish T2DM patients without known coronary artery disease or overt heart disease (21), the prevalence of Grade 2 LVDD was slightly higher (18%) and total LVDD lower (40%) than our findings. These patients had a mean age like ours but a shorter mean duration of T2DM (4.5 years) that may account for its lower prevalence compared to our cohort. Only 9% of the Danish cohort had LVSD. There were also no details of therapy provided. In a French cohort of T2DM patients (15) (mean duration of 11 years), which excluded patients with EF < 55% and coronary artery disease (diagnosed by stress testing/myocardial perfusion studies within 1 month of enrolment), the prevalence of LVDD was 47% (33% Grade 1 and 14% Grade 2). The lower prevalence of LVDD compared to our cohort can be explained by younger patients and the exclusion of those with coronary artery disease using functional and imaging modalities. However, it is surprising that despite the long duration of T2DM and lack of RAS blockers use, there was a relatively low prevalence of LVDD.

Population characteristics and healthcare system practices can determine prevalence of HF and LVDD. In a cross-sectional study (22) of 581 Dutch patients with T2DM without known HF, 27.7% were diagnosed as having HF during the study duration (22.9% with HFpEF and 4.8% with HFrEF). One in four had asymptomatic LVDD and 0.7% asymptomatic LVSD. The alarmingly high rates of HFpEF in this population may be accounted for by an older cohort (as age is associated with LVDD), the low usage of RAS blockers, and the inclusion of patients with IHD. These patients (unlike ours) were recruited from a primary care setting, which may have influenced medication use; e.g., only 52.7% were on RAS blockers although 65.6% had hypertension.

Our patients were not known to have and were asymptomatic of CV disease. Absence of regional wall motion abnormalities in all study patients suggested they did not have coronary artery disease; however, we did not perform coronary angiogram to ascertain it. This coupled with tertiary healthcare where almost three-fourths of our patients were on RAS blockers, >90% on statins, and 41.9% on SGLT2-i may account for the low prevalence of HF, LVSD, and asymptomatic high-grade LVDD. Unlike our work, none of the forementioned studies, Asian or Western, were carried out in the current T2DM management landscape where SGLT2-i use is widespread.

Reported associations of LVDD in patients with T2DM are age, female gender, duration of T2DM, HbA_1c_ status, obesity, higher systolic blood pressure, and presence of albuminuria, CKD ≥ Stage 3, and retinopathy (1, 15, 17, 19, 21, 23–26). Upon univariate analysis, we found that age, hypertension, hyperlipidemia, insulin therapy, and ethnicity were correlated with presence of asymptomatic LVDD. However, upon multiple logistic regression, only older age, insulin therapy, and Chinese ethnicity were confirmed to be independently, positively associated. Duration of T2DM was not an independent predictor of LVDD. This may be due to skewing of our tertiary healthcare center population toward those with long-standing T2DM, with >80% having been diagnosed for >10 years. On the other hand, insulin therapy was independently correlated with presence of LVDD. T2DM has a long preclinical asymptomatic stage; hence, the point of diagnosis does not truly reflect the true onset of disease. The natural history of T2DM is characterized by progressive loss of β-cell function over the years, leading to secondary sulfonylurea failure and the eventual need for insulin therapy (27, 28). Therefore, the need for insulin therapy in T2DM may be a more accurate surrogate marker of long-duration T2DM. Our findings are similar to those of Nichols et al. who also found insulin therapy to be independently associated with HF in patients with T2DM (29).

We found patients of Chinese ethnicity to be at higher risk of LVDD. Subgroup analysis of these patients did not find any difference in glycemic control, hypertension, retinopathy, albuminuria, renal function, and SGLT2-i or antihypertensive therapy. These patients however were significantly older, with a lower BMI and a lower proportion of insulin use. Indians, who originate from South Asia, are known to have higher risk for atherosclerotic CV disease, while East Asians have a lower risk (7). Our study excluded patients with known CV disease, which probably led to Indian ethnicity being not a risk for LVDD. East Asians may be at higher risk of LVDD potentially due to unelucidated genetic factors. Our findings need to be validated and further evaluated in larger sample populations that include Chinese and other East Asian ethnic groups.

Similar to the SHORTWAVE study, we did not find glycemic control to be independently associated with asymptomatic LVDD (1). This is in contrast with findings from previous studies which found a positive correlation between HbA_1c_ and echocardiographic parameters of LVDD (19) and incident HF (30, 31). Iribarren et al. observed that a 1% increase in HbA_1c_ was associated with an 8% increased risk of HF in a large cohort of 50,000 patients with diabetes (30). When interpreting our results in the light of these past studies, we should consider that the positive associations between HbA_1c_ and incident symptomatic HF may not be applicable in our patients with asymptomatic LVDD. These studies were also conducted in populations that were managed prior the usage of RAS blockers and statins as standard of care. The analysis by Iribarren et al. was conducted in a cohort where 11% of those with HF had coronary artery disease at diagnosis (30). Also, the evaluated cohort had only 15 and 26% of its population treated with insulin and ACE-I, respectively, and 23% with HbA_1c_ < 7%, while 71% did not have low-density lipoprotein cholesterol (LDL-C) levels reported. Therefore, a comparison with our cohort of patients who did not have coronary artery disease and were managed with a vastly different standard of care protocol cannot be equivalent. In another prospective population-based study (31), a cohort of 1,827 participants of the Atherosclerosis Risk in Communities study found similarly strong association between baseline HbA_1c_ and incident HF after a mean of 9.9 years' follow-up. No specific data on ACE-i and statin use were reported in this paper, and risk of HF was not adjusted for use of these medications. Neither BMI nor obesity was associated with presence of LVDD on univariate or multivariate analysis in our sample population, which is similar to the findings from the SHOCKWAVE cohort (1).

Asymptomatic LVDD in patients with T2DM is likely due to microangiopathy, interstitial fibrosis, extracellular collagen deposition, calcium transport abnormalities, and neurohormonal alterations and is the earliest manifestation of diabetic cardiomyopathy (2, 15). Presence of asymptomatic LVDD has been associated with higher risk of progression to atrial fibrillation, HF, and all-cause mortality in patients with T2DM (2, 23, 24, 32). Although presence of Grade 1 LVDD in the majority of our cohort may not seem very alarming, evidence from prospective cohort studies in Western populations (2, 33) indicates that even patients with Grade 1 LVDD can deteriorate with time, with the main predictors being aging, retinopathy, and increase in blood pressure. In patients with T2DM and asymptomatic LVDD, robust analyses have shown that adjusted risk of progression to HF is increased by 61% compared with those without LVDD, with a 5-year cumulative probability of developing HF of 36.9% (2).

Given that the presence of LVDD and its potential for progression have important prognostic implications, our findings of high prevalence of mild LVDD indicate the need for strategies to screen for and monitor progression of LVDD with regular ECHO, clinical examination, and appropriate preventive measures to control modifiable risk factors such as hypertension, hyperlipidemia, retinopathy, progressive nephropathy, and improvement of glycemic control.

## Conclusion

This cross-sectional study demonstrated a high prevalence (70.1%) of asymptomatic LVDD in a high-risk cohort of patients with T2DM treated with RAS blockers, statins, and SGLT2-I, without prior known CV disease. Most of the LVDD patients were at Grade 1/mild severity. Older age, insulin therapy, and Chinese ethnicity were risk factors for diastolic dysfunction. Such findings emphasize the need for regular screening and monitoring for progression of cardiac dysfunction as well as appropriate therapeutic risk mitigation measures given the long-term prognostic implications of LVDD.

## Data Availability Statement

The raw data supporting the conclusions of this article will be made available by the authors, without undue reservation.

## Ethics Statement

The studies involving human participants were reviewed and approved by University of Malaya Research Ethics Committee (UMREC). The patients/participants provided their written informed consent to participate in this study.

## Author Contributions

KHC, ATBT, and KLT conceived the original study concept and contributed to the study design. KLT, IL, SSS, YYC, and KC participated in data collection and analysis. KHC and KLT drafted the manuscript with input from all authors. All authors met the International Committee of Medical Journal Editors criteria for authorship and have read and approved the final manuscript.

## Conflict of Interest

The authors declare that the research was conducted in the absence of any commercial or financial relationships that could be construed as a potential conflict of interest.

## Publisher's Note

All claims expressed in this article are solely those of the authors and do not necessarily represent those of their affiliated organizations, or those of the publisher, the editors and the reviewers. Any product that may be evaluated in this article, or claim that may be made by its manufacturer, is not guaranteed or endorsed by the publisher.
